# Next-Generation Therapeutics for IBD

**DOI:** 10.1007/s11894-015-0444-2

**Published:** 2015-06-02

**Authors:** Mark Löwenberg, Geert D’Haens

**Affiliations:** Department of Gastroenterology and Hepatology, Academic Medical Center, Amsterdam, The Netherlands; Robarts Clinical Trials, Amsterdam, The Netherlands

**Keywords:** Crohn’s disease (CD), Inflammatory bowel disease (IBD), Integrin inhibitors, Small molecules, Tofacitinib, Ulcerative colitis (UC), Vedolizumab

## Abstract

Various novel drugs have recently been evaluated in clinical trials showing promising effects in patients with inflammatory bowel disease (IBD). Here, we summarize the recent literature in the area of emerging therapies in the field of IBD, with specific focus on anti-integrin antibodies, such as vedolizumab (anti-α4β7) and etrolizumab (anti-rhuMAb β7), and the Janus kinase (JAK) inhibitor tofacitinib. Moreover, we will discuss efficacy and safety data of golimumab (a new subcutaneous anti-tumor necrosis factor (TNF) antibody), Avaxia (an orally delivered anti-TNF antibody), and Budesonide MMX; all have been developed for the treatment of ulcerative colitis. Other therapeuticals that might find their way to the market the coming years include the anti-mucosal vascular addressin cell adhesion molecule (MAdCAM) PF-00547659, small molecules (including laquinimod and the CCR9 antagonist Vercirnon), as well as an orally active SMAD7 antisense oligonucleotide that showed clinical benefit in Crohn’s disease patients.

## Introduction

Inflammatory bowel diseases (IBD) are chronic inflammatory disorders of the gastrointestinal tract that comprise Crohn’s disease (CD) and ulcerative colitis (UC). Advances in understanding the pathogenesis of these conditions have led to the development of new therapies. The most important progress in the management of IBD patients during the last 15 years has been the introduction of anti-tumor necrosis factor (TNF) agents [1–4, 5••]. However, failure to TNF blockers is frequently observed, a problem that complicates clinical management. Up to 30 % of IBD patients do not respond to induction therapy with anti-TNF agents (primary non-response) [6, 7], and a significant proportion loses response over time (secondary non-response) [8]. As a consequence, approximately one third of patients are in clinical remission 1 year after initiation with anti-TNF agents. Loss of response to infliximab and adalimumab was calculated to be 13 and 25 % per year, respectively [9, 10]. Recent work showed that fecal loss of infliximab is associated with primary non-response in IBD patients (Brandse J et al., abstract presentation DDW 2013, Gastroenterology, Volume 144, Issue 5, S-36; Table 1). Higher infliximab doses are likely needed as induction therapy in case of severe colitis to compensate for fecal loss of infliximab.

**Table 1:** Oral presentations

Brandse J et al.	Oral presentation DDW 2013
Harris M et al.	Oral presentation UEGW 2014
Sands B et al.	Poster presentation UEGW 2014
Hanauer S et al.	Poster presentation UEGW 2014
Feagan B et al.	Poster presentation UEGW 2014
Feagan B et al.	Oral presentation CCFA 2013
Monteleone G et al.	Oral presentation UEGW 2014

Anti-TNF agents are generally well tolerated, but their use is linked to safety issues, including risk for infections and malignancies [11–13]. Hence, there remains an unmet need for new treatment options for these patients. Several novel drugs showed potent clinical effects in IBD trials. They include small molecules interfering with intracellular signaling pathways and therapeutic antibodies that are directed against extracellular targets, such as pro-inflammatory cytokines or receptors. An orally administered antisense nucleotide against SMAD7 (GED-301) was also used successfully for active CD. This review article aims to review efficacy and safety data from clinical trials with integrin blockers, small molecules, oral nucleotide therapy against SMAD7, new anti-TNF compounds, and a novel corticosteroid (Budesonide MMX: Cortiment® and Uceris®). We will also focus on the potential positioning of the approved agents in the therapeutic armamentarium of IBD.

## Novel Anti-TNF Agents: Golimumab and Avaxia

Golimumab (Simponi®), a human monoclonal antibody against TNF, was recently approved by the US Food and Drug Administration (FDA) and the European Medicines Agency (EMA) for UC patients with moderate to severe active disease [14]. Regulatory approval was based on the results of the Program of Ulcerative Colitis Research Studies Utilizing an Investigational Treatment (PURSUIT) induction and maintenance trial [5••, 15••]. Induction treatment with golimumab consists of 200 and 100 mg subcutaneously injected at weeks 0 and 2. Maintenance treatment is given at doses of 100 mg every 4 weeks in the USA and 50 mg every 4 weeks in European jurisdictions, except for patients with a body weight of more than 80 kg where 100 mg is recommended.

Induction treatment with golimumab in patients with moderate to severe active UC failing standard treatment but naïve to biologic treatment resulted in significantly greater clinical response, clinical remission, and mucosal healing rates at week 6 compared to placebo [5••]. Clinical response rates at week 6 (primary endpoint of the induction trial) were 51.0, 54.9, and 30.3 % of patients receiving golimumab 200/100 mg, 400/200 mg, or placebo (*p* < 0.0001 for both golimumab groups versus placebo), respectively. Clinical remission rates were seen in 17.8, 17.9, and 6.4 % of patients receiving golimumab 200/100 mg, 400/200 mg, or placebo (*p* < 0.0001 for both comparisons versus placebo). Mucosal healing, defined as a Mayo endoscopic score of ≤1 at week 6, was seen in 45.1, 42.3, and 28.7 % of patients receiving 400/200 mg golimumab, 200/100 mg golimumab, or placebo, respectively (*p* < 0.05 for both comparisons versus placebo). Patients who responded to golimumab induction therapy were enrolled in the PURSUIT maintenance trial and were randomized to receive 50 or 100 mg golimumab every 4 weeks or placebo [15••]. Patients who did not respond to golimumab induction treatment received open-label treatment with 100 mg golimumab every 4 weeks. A novel clinical endpoint, “continued clinical response” (CCR) defined as clinical response at all study visits, was used in this trial for the first time. CCR up to 54 weeks was seen in 47.0 and 49.7 % of patients receiving 50 or 100 mg golimumab every 4 weeks, respectively, compared to 31.2 % in the placebo group (*p* = 0.010 and *p* < 0.001, respectively). At week 30 and week 54, a higher percentage of patients in the 100 mg golimumab group were in clinical remission and had mucosal healing (27.8 and 42.4 %, *p* = 0.004) versus placebo (15.6 and 26.6 %, *p* = 0.002) or 50 mg golimumab (23.2 and 41.7 %, respectively). Golimumab induction and maintenance treatment was well tolerated without new safety signals for this anti-TNF agent and with surprisingly few local injection reactions and immunogenicity. The decision which anti-TNF agent is recommended for the individual UC patient needs further study, but it appears that the subcutaneous agents are less potent than intravenous infliximab, which should be the preferred treatment modality for severe and certainly hospitalized patients.

Avaxia Biologics (Lexington, MA, USA) developed an orally administered anti-TNF antibody (AVX-470), isolated from colostrum of dairy cows that had been immunized with TNF, and tested the drug in patients with acute moderate to severe UC (Harris M et al., abstract presentation UEGW 2014). Safety (primary endpoint) as well as pharmacokinetics, immunogenicity, and efficacy (e.g., clinical and endoscopic response and remission) were evaluated in 36 patients. Subjects received active drug (0.2, 1.6, or 3.5 g/day) or placebo for four consecutive weeks. The drug was well tolerated. Twenty-eight percent of the patients receiving active treatment with AVX-470 showed a clinical response versus 14.3 % in the placebo group. Dose-related improvements in clinical and endoscopic remission were seen at higher doses. Future studies will have to assess the effects of higher doses and longer duration of treatment.

## Budesonide MMX (Uceris® and Cortiment®)

The use of systemic corticosteroids is associated with many adverse effects. Budesonide is a corticosteroid with reduced systemic activity due to limited resorption and high first-pass metabolism. Budesonide MMX (available as Cortiment® by Ferring in Europe and Uceris® by Santarus Inc. in the USA) is a novel once-daily oral formulation that uses colonic-release multi-matrix system (MMX) technology allowing controlled release of the drug into the colon [16, 17]. A placebo-controlled study compared the efficacy and safety of Budesonide MMX in patients with mild to moderate active UC [17]. Patients were randomized to receive Budesonide MMX (9 or 6 mg/day), Entocort 9 mg/day (e.g., budesonide controlled ileal-release capsules; AstraZeneca, Södertälje, Sweden), or placebo once daily for 8 weeks. Combined clinical and endoscopic remission rates (primary endpoint) with Budesonide MMX 9 mg, Budesonide MMX 6 mg, Entocort, and placebo were 17.4, 8.3, 12.6, and 4.5 %, respectively (*p* = 0.0047 for Budesonide MMX 9 mg versus placebo).

A second trial compared Budesonide MMX with mesalamine and placebo for induction of remission in patients with mild to moderate active UC [18]. Patients were randomly assigned to receive Budesonide MMX (9 or 6 mg/day), mesalamine (2.4 g/day), or placebo for 8 weeks. Clinical remission rates at week 8 (primary endpoint) were 17.9, 13.2, and 12.1 % in the 9-mg Budesonide MMX, 6-mg Budesonide MMX, or mesalamine group, respectively, compared with 7.4 % in the placebo group (*p* = 0.0143, *p* = 0.1393, and *p* = 0.2200 for the three comparisons versus placebo). No significant differences in adverse events were seen between Budesonide MMX- and placebo-treated patients in these two trials (CORE I and II), but the follow-up period was only 8 weeks. Hence, induction treatment with Budesonide MMX (9 mg once daily) is significantly more effective in achieving clinical and endoscopic remission versus placebo in patients with mild to moderately active UC. The agent is currently approved both in Europe (as Cortiment) and in the USA (as Uceris®).

At this moment, Budesonide MMX should be considered as second-line treatment in patients with mild to moderate UC who fail optimal mesalamine therapy and before the use of systemic corticosteroids. Since no studies have evaluated efficacy beyond a treatment period of 8 weeks, Budesonide MMX is not recommended as maintenance treatment. Since only one dose is available (9 mg/day), a tapering regimen does not appear to be necessary following successful induction.

## Agents Interfering With Leukocyte Trafficking

The pathogenesis of IBD is characterized by leukocyte infiltration into the gut which is mediated by chemokines and the interaction between integrins (located on the cell surface of lymphocytes) and their specific ligands on gut endothelial cells. Drugs that selectively target cell adhesion molecules interfere with T cell trafficking. Natalizumab (Tysabri® or Antegren®) was the first therapeutic antibody that was tested in humans with multiple sclerosis [19] and CD [20] and blocks the α4 integrin on lymphocytes constituting immune surveillance in the central nervous system and the gut. Natalizumab induced and maintained remission in patients with moderate to severe CD, but was found to carry an increased risk of progressive multifocal leukoencephalopathy (PML), a central nervous system JC virus infection that can be lethal due to impaired central nervous system immune surveillance. Presence of anti-JC virus antibodies, use of immunosuppressive agents, and increased duration of natalizumab treatment are associated with PML risk [21]. The incidence of this infection is estimated at about 1/300. Shortly after its approval by the FDA, natalizumab was temporarily withdrawn from the market but was then reintroduced in 2006 in the USA using a surveillance program and only in patients without other immunomodulator therapy. The next-generation anti-adhesion molecules, including vedolizumab, etrolizumab, and PF-00547659, are more gut selective and have therefore not led to PML, so far.

### Vedolizumab

Vedolizumab (Entyvio®) is the first gut-selective humanized monoclonal antibody. This drug was approved in 2014 by the FDA and EMA for both UC and CD, refractory to standard therapy and/or anti-TNF agents. In contrast to natalizumab, which lacks selectivity for the gut, vedolizumab specifically targets α4β7 integrins that is exclusively present on gut-homing T cells thereby blocking the interaction between α4β7 and anti-mucosal vascular addressin cell adhesion molecule (MAdCAM)-1.

GEMINI I was a double-blind phase 3 trial in patients with moderate to severe UC [22••]. Patients were randomized to receive vedolizumab (300 mg intravenously) or placebo on day 1 and day 15. Clinical response at week 6, the primary endpoint of the induction trial, was achieved in 47 % of patients receiving vedolizumab compared to 26 % on placebo (*p* < 0.0001), and clinical remission at week 6 was seen in 17 % on vedolizumab versus 5 % on placebo (*p* = 0.0009). Mucosal healing rates were 41 and 25 % of patients treated with vedolizumab versus placebo (*p* = 0.0012). Patients who achieved a clinical response after induction therapy were randomized to receive placebo or further intravenous vedolizumab at 300 mg at 4- or 8-week dosing intervals up to 46 weeks. Significantly greater clinical remission rates were seen in patients receiving vedolizumab versus placebo at week 52 (42 and 45 % in the vedolizumab 8- and 4-weekly groups, respectively, versus 16 % in the placebo arm; *p* < 0.0001 for both comparisons versus placebo). Moreover, mucosal healing rates were significantly higher in the vedolizumab group compared to placebo (52 and 56 % in the vedolizumab 8- and 4-weekly groups, respectively, versus 20 % in the placebo group; *p* < 0.0001). Of note, the overall clinical efficacy was higher with vedolizumab in anti-TNF-naïve patients than in subjects with prior failure or intolerance to anti-TNF agents. Clinical remission rates at week 52 were 45 versus 18 % and 37 versus 5 % in UC patients who were naïve and exposed to prior anti-TNF treatment, respectively.

Clinical response at week 6 was also the primary endpoint of the induction trial with vedolizumab in patients with moderate to severe CD (GEMINI II) [23••]. Clinical remission at week 6 was seen in 13.3 versus 9.7 % of anti-TNF failures on vedolizumab and placebo, respectively (*p* = 0.157). Corresponding remission rates were 22.7 versus 10.6 % of patients who were naïve for anti-TNF agents receiving treatment with vedolizumab or placebo (*p* = 0.005). At week 10, clinical remission rates were 21.7 versus 11 % in anti-TNF failures on vedolizumab or placebo (*p* = 0.0008), respectively, and 24.7 versus 15.4 % in anti-TNF-naïve patients receiving vedolizumab or placebo (*p* = 0.044). Week 52 clinical remission rates were 52 and 27 % in vedolizumab and placebo-treated patients who were naïve for anti-TNF agents. In contrast, the clinical response rate at week 52 was lower after anti-TNF failure: 28 versus 13 % in the vedolizumab and placebo groups, respectively (35 % of vedolizumab-treated CD patients had received ≥2 anti-TNF agents) [23••]. Recent work suggested that certain patients benefited from an increase in vedolizumab dosing frequency from every 8 weeks to every 4 weeks in GEMINI I (UC) or GEMINI II (CD) during the open-label long-term extension study (GEMINI LTS) (poster presentation UEGW 2014, P1054. Sands B et al.). Overall, the results with vedolizumab seem to be somewhat better in UC compared to CD. Most likely, the 6-week time point in CD was set too early to appreciate optimal efficacy given the mode of action of this agent. It is recommended that CD patients that do not show response at week 10 be given an additional 300-mg infusion at week 10. In the absence of effect at week 14, it appears logical to discontinue treatment at that point and consider alternative options.

A placebo-controlled phase 3 trial evaluated efficacy and safety of vedolizumab in CD patients who had failed anti-TNF therapy (GEMINI III). In the anti-TNF failure population, 15.2 and 12.1 % of patients receiving vedolizumab or placebo were in clinical remission at week 6, respectively (*p* = 0.433). However, vedolizumab was statistically superior to placebo for inducing clinical remission at week 10 (secondary outcome parameter). Clinical remission at week 10 was seen in 26.6 % of patients in the vedolizumab group versus 12.1 % in the placebo arm (*p* = 0.001) [24••].

The 2-year efficacy data of vedolizumab in CD (poster presentation UEGW 2014, P1059, Hanauer S et al.) and in UC (poster presentation UEGW 2014, P1667, Feagan B et al.) were recently presented. Patients with UC (GEMINI I) and CD (GEMINI II) who completed 52 weeks of vedolizumab treatment were enrolled in GEMINI LTS showing long-term efficacy and safety for an additional 52 weeks. Hence, the safety profile of vedolizumab in the GEMINI program is reassuring. To date, there have been no cases of PML, and the incidence of systemic and gastrointestinal infections was similar among patients on vedolizumab or placebo. Recently, a study has been conducted in order to determine whether vedolizumab alters T cell subpopulations in cerebrospinal fluid. Samples of cerebrospinal fluid were obtained from healthy volunteers and analyzed by flow cytometry before and after treatment with vedolizumab. No significant changes in T cell populations were observed [25].

Among pooled UC and CD patients from GEMINI I and II, only 4 % tested positive for anti-vedolizumab antibodies during maintenance treatment with vedolizumab. Similar rates of anti-drug antibodies were observed in the vedolizumab monotherapy (only 3 % of patients) and vedolizumab-immunomodulator combination (4 % had increased anti-vedolizumab antibody titers) groups. The presence of anti-vedolizumab antibodies might have clinical implications, but further studies are needed to look into this question. An important question to be answered is how vedolizumab should be positioned in future treatment algorithms. Given its higher efficacy in TNF-naïve patients and the favorable safety profile, the drug has the potential to become the first line biologic in UC.

### Etrolizumab

The humanized monoclonal antibody etrolizumab (rhuMAb β7) interferes with leucocyte migration by selectively targeting the β7 subunit of α4β7 and αEβ7 integrins. In a placebo-controlled randomized phase 2 trial, patients with moderate to severe UC received subcutaneous etrolizumab (100 mg at weeks 0, 4, and 8, with placebo at week 2 or 420 mg at week 0 and 300 mg etrolizumab at weeks 2, 4, and 8) or placebo [26•]. A significant greater number of patients treated with the study drug achieved clinical remission at week 10 (primary endpoint) compared to placebo. The primary endpoint was met by 21 and 10 % of patients in the 100- or 300-mg-etrolizumab group, respectively, and in 0 % of patients receiving placebo. This low placebo response rate was probably a consequence of careful patient selection (patients were included with a Mayo endoscopic subscore ≥2 using central endoscopic reading) and stringent endpoints. Biopsy analysis in this trial revealed that αE expression could be a suitable biomarker to identify patients that are most likely to benefit from etrolizumab treatment [26•]. It was shown that increased αE expression levels in the inflamed colon were associated with enhanced clinical benefit of etrolizumab treatment in UC. High αE expression levels in baseline biopsies, analyzed with qPCR and immunohistochemistry, were enriched for etrolizumab remission at 10 weeks, with a higher enrichment in anti-TNF-naïve patients. Future trials should further characterize the potential role of αE as a biomarker and should focus on long-term follow-up. Larger phase 3 studies have now been initiated including a head-to-head comparison versus adalimumab in UC and additional trials in CD.

### PF-00547659

A randomized placebo-controlled trial investigated safety and efficacy of PF-00547659, a monoclonal antibody against MAdCAM-1, in patients with active UC [27•]. In total, 80 patients were enrolled receiving a single or three doses of PF-00547659 (0.03–10 mg/kg, intravenously or subcutaneously administered) or placebo at 4-week dosing intervals. Adverse events were reported in similar proportions of patients on PF-00547659 versus placebo, the most common adverse event being abdominal pain. PF-00547659 produced some potential benefits compared to placebo on clinical and endoscopic efficacy endpoints, but no statistical differences were found between actively treated patients and placebo. Clinical response at week 4 was seen in 32 and 52 % of patients on placebo or PF-0054659 (all doses) (*p* = 0.102). Week 12 clinical response rates were 21 versus 42 % in the placebo and PF-00547659 groups, respectively (*p* = 0.156). Endoscopic improvement was higher, but not significantly different, in patients receiving PF-00547659 compared to placebo. Larger clinical trials evaluating efficacy of PF-00547659 in CD and UC will be completed in 2015. The safety of this agent regarding central nervous system infections appeared reassuring in the TOSCA trial, an open-label study in which patients with active CD had lumbar punctures before and after anti-MAdCAM treatment. No changes in cerebrospinal fluid cell populations were detected (D’Haens G et al., abstract presentation ECCO 2014, OP007).

### Anti-CCR 9 (Vercirnon)

The chemokine receptor CCR9 plays an important role in the process of gut homing of leucocytes. CCX282-B, also called Vercirnon or Traficet-EN (ChemoCentryx, Inc., CA, USA), is a chemokine receptor CCR9 antagonist that is orally administered. This drug interferes with migration and activation of inflammatory cells in the intestine [28•]. Recently, safety and efficacy of this drug were evaluated in more than 400 CD patients who received placebo or CCX282-B (250 or 500 mg once daily or 250 mg twice daily) for 12 weeks, followed by 250 mg CCX282-B twice daily up to week 16 (open-label) [28•]. Week 16 clinical responders were randomized to receive CCX282-B (250 mg twice daily) or placebo for 36 weeks. Response rates at week 8 were 49 % in the placebo group, 52 % in the CCX282-B 250 mg once daily group (*p* = 0.667 versus placebo), 48 % in the CCX282-B 250 mg twice daily group (*p* = 0.833), and 60 % in patients receiving once-daily CCX282-B 500 mg (*p* = 0.111). Remission rates at week 52 were seen in 47 % of patients on CCX282-B versus 31 % of patients on placebo (*p* = 0.012); 46 % showed a sustained clinical response compared to 42 % in the placebo arm (*p* = 0.629). Treatment with this small-molecule inhibitor was well tolerated. The initial trial by ChemoCentryx was followed by a large phase 3 program in CD sponsored by GlaxoSmithKline. Surprisingly, the initial results could not be reproduced. The trial was negative without even a hint of efficacy of CCX282-B. The program was immediately discontinued (Feagan B et al., abstract presentation CCFA 2013).

## Novel Small Molecules

Orally active small molecules interfere with intracellular signaling. Compared to therapeutic antibodies, small molecules have certain advantages, including lower production costs and usually oral route of administration. Most advanced in their development are the Janus kinase (JAK) inhibitors, one of which is already approved and marketed for the treatment of rheumatoid arthritis in the USA.

### Tofacitinib

JAKs play a key role in regulating cellular proliferation, differentiation, and immune cell functions. They can convert extracellular signals (cytokines, growth factors, etc.) into genomic responses. JAK-dependent intracellular signaling pathways are involved in the pathophysiology of many chronic inflammatory disorders, including rheumatoid arthritis and IBD [29–31]. Hence, JAK inhibitors have become attractive anti-inflammatory agents although they carry the risk of profound systemic immunosuppression.

Tofacitinib inhibits JAK1 and JAK3 signaling resulting in reduced synthesis of pro-inflammatory cytokines. A phase 2 trial in 194 patients with moderate to severe active UC assessed safety and efficacy of tofacitinib at doses of 0.5, 3, 10, or 15 mg or placebo twice daily for 8 weeks [32••]. Clinical response at week 8 (primary outcome) was seen in 32, 48, 61, and 78 % of patients receiving tofacitinib at a dose of 0.5 mg (*p* = 0.39), 3 mg (*p* = 0.55), 10 mg (*p* = 0.10), and 15 mg (*p* < 0.001), respectively, as compared with 42 % of patients in the placebo group. Week 8 clinical remission was observed in 13, 33, 48, and 41 % of patients receiving tofacitinib at doses of 0.5 mg (*p* = 0.76), 3 mg (*p* = 0.01), 10 mg (*p* < 0.001), and 15 mg (*p* < 0.001), respectively, versus 10 % of patients in the placebo group. Endoscopic remission at 8 weeks (defined as a Mayo endoscopy subscore of 0) was seen in 2 % of patients receiving placebo, compared to 10 % receiving 0.5 mg tofacitinib (*p* = 0.14), 18 % receiving 0.5 mg tofacitinib (*p* = 0.01), 30 % receiving 10 mg tofacitinib (*p* < 0.001), and 27 % receiving 15 mg tofacitinib (*p* < 0.001). Two phase 3 registration trials are ongoing.

Tofacitinib was also evaluated in patients with moderate to severe active CD [33•]. Patients were randomized to receive tofacitinib twice daily for 4 weeks at doses of 1 mg, 5 mg, 15 mg, or placebo. Clinical response rates at week 4 (primary endpoint) were 36 % (*p* = 0.467), 58 % (*p* = 0.466), and 46 % (*p* ≥ 0.999) of patients given 1, 5, or 15 mg tofacitinib twice daily versus 47 % of patients given placebo. Clinical remission at week 4 (secondary endpoint) was seen in 31 % (*p* = 0.417), 24 % (*p* = 0.776), and 14 % (*p* = 0.540) of patients given 1, 5, and 15 mg tofacitinib, versus 21 % of patients given placebo. Strictly, this trial was negative, but the placebo response and remission rates were unexpectedly high. A reduction in fecal calprotectin and C-reactive protein levels among patients receiving 15 mg tofacitinib twice daily indicated its biologic activity. A repeat phase 2 trial with stricter inclusion criteria defining active disease is ongoing. Meanwhile, the maximum dose of 15 mg twice daily has been abandoned given the lack of superiority over 10 mg twice daily.

Tofacitinib seems to have an acceptable safety profile in UC and CD patients, although herpes zoster infections have been observed quite frequently. In rheumatoid arthritis, several cases of lymphoma were reported, but the overall risks of infections and mortality with tofacitinib seem to be similar to those observed with biologicals [34]. In addition, a dose-dependent increase in low-density and high-density lipoprotein cholesterol concentrations occurred. It is unclear what the long-term effects of this phenomenon could be. The most attractive property of the JAK inhibitors is that they can be used as monotherapy and that they are orally available. Other JAK inhibitors are currently under clinical investigation in phase 2 for both CD and UC.

### Laquinimod

Laquinimod is an oral immunomodulator that induces anti-inflammatory effects by modulating immune cells resulting in reduced synthesis of several cytokines. The agent was effective in suppressing disease activity in multiple sclerosis and seems to have a favorable safety profile [35]. Laquinimod is also in development for the treatment of CD. Recently, a phase 2a trial was published in which patients with active CD received different doses of laquinimod (0.5, 1, 1.5, or 2 mg/day) for 8 weeks [36•]. Clinical remission at week 8 was seen in 48.3, 26.7, 13.8, and 17.2 % of patients receiving 0.5, 1, 1.5, and 2 mg laquinimod versus 15.9 % in the placebo group. Interestingly, clinical benefit was most pronounced at week 8 with the lowest dose whereas the 1.5- and 2-mg doses appeared ineffective. Overall, induction treatment with laquinimod was reasonably well tolerated with headache, abdominal pain, nausea, vomiting, and musculoskeletal pain as the most common adverse effects. All laquinimod doses were found to reduce fecal calprotectin levels in these patients. Further studies with this small molecule are warranted.

## Antinucleotide Therapy

### SMAD7 Antisense (Mongersen, GED-301)

The safety and efficacy of Mongersen, an orally administered SMAD7 antisense oligonucleotide (Celgene, Switzerland), were recently studied in a placebo-controlled phase 2 study in patients with active CD (Monteleone G et al. abstract presentation UEGW 2014, OP203). SMAD7 is an endogenous inhibitor of the immunosuppressive cytokine transforming growth factor-β1. Patients were randomized to receive induction treatment with different doses of Mongersen or placebo for 2 weeks. Clinical remission (primary outcome parameter) was seen in 55.0, 65.1, and 9.5 % of patients receiving Mongersen 40 mg/day, 160 mg/day, or placebo (*p* < 0.0001, for both comparisons) at 15 days and maintained for ≥2 weeks. Adverse events were similar across the treatment groups. This promising molecule is now being investigated in larger trials.

## Conclusion

Anti-TNF agents have been on the market for more than 15 years and have found an established place in the therapeutic armamentarium, primarily moderate to severe UC and CD. In CD, evidence suggests that earlier introduction leads to superior outcomes and higher rates of mucosal healing. The latest addition to the anti-TNF class of drugs is golimumab, a subcutaneous human anti-TNF agent that was recently approved by the FDA and EMA for the treatment of moderately to severely active UC. Given the fact that only one third of CD and UC patients show a durable response to all available anti-TNF agents, there remains an unmet need for novel treatment options. Oral TNF blockers could offer an attractive option.

Recent trials showed that the gut-selective integrin inhibitor vedolizumab (an antibody that blocks α4β7-MAdCAM-1 interactions) is efficacious for UC and CD, but the results seem to be better in UC. Other adhesion molecule blockers (including etrolizumab and PF-00547659) also showed promising clinical effects in UC but need further trials. The anti-integrin antibodies appear to be well tolerated, although long-term efficacy and toxicity data need to be collected.

Various small molecules that interfere with inflammatory pathways are currently under development or are being evaluated in clinical trials. Tofacitinib has shown promising clinical effects in UC and is in phase 3. Other JAK inhibitors are in the therapeutic pipeline and might find their way to the market as well. Laquinimod may hold promise for CD. Finally, Mongersen (GED-0301), a SMAD7 antisense nucleotide with magnificent phase 2 results, will soon enter phase 3. The question is how these novel agents will be positioned in future IBD treatment algorithms. Vedolizumab has the potential to become a first-line biologic for the treatment of UC. The JAK inhibitor tofacinitib may also appear on the market for UC in the foreseeable future, depending on the outcome of the ongoing phase 3 trials. Looking at the wide range of molecules in the development for IBD, the therapeutic options in these conditions will improve to an important extent and the positioning of the conventional agents is likely to change significantly.
